# Extracellular amoebal-vesicles: potential transmission vehicles for respiratory viruses

**DOI:** 10.1038/s41522-021-00201-y

**Published:** 2021-03-17

**Authors:** Rafik Dey, Melanie A. Folkins, Nicholas J. Ashbolt

**Affiliations:** 1 School of Public Health, University of Alberta, Edmonton, AB Canada; 2 Dept. Medical Microbiology and Immunology, University of Alberta, Edmonton, AB Canada; 3 School of Environment, Science and Engineering, Southern Cross University, Lismore, NSW Australia

**Keywords:** Water microbiology, Biofilms, Pathogens

## Abstract

Human respiratory syncytial virus (RSV) is a major cause of acute respiratory tract infections in children and immunocompromised adults worldwide. Here we report that amoebae-release respirable-sized vesicles containing high concentrations of infectious RSV that persisted for the duration of the experiment. Given the ubiquity of amoebae in moist environments, our results suggest that extracellular amoebal-vesicles could contribute to the environmental persistence of respiratory viruses, including potential resistance to disinfection processes and thereby offering novel pathways for viral dissemination and transmission.

Amoebae are amongst the most ubiquitous organisms in natural and engineered environments^1–3^. They live at interfaces (water-soil, water-animal, water-plants and water-air), adherent on various surfaces and feed on microorganisms^3^. While relatively few amoebae species are pathogens in their own right^4^, they are known natural environmental reservoirs for a range of amoeba-resisting bacterial pathogens, such as *Legionella pneumophila*, a water-based bacterium responsible for Legionnaires’ disease that results in major community health burden^5–8^. More recently, amoebae have been identified as potential reservoirs for non-enveloped respiratory and enteric viruses such as adenoviruses, coxsackieviruses, reovirus and the giant amoeba virus *Mimivirus*^9–12^. Several highly transmissible respiratory enveloped viruses with epidemic potential have emerged in last two decades, with the ongoing COVID-19 pandemic being the most significant to date^13^, yet their potential interaction with (sewage/faecal-borne) amoebae is unreported.

Human respiratory syncytial virus (RSV) is a large (120–300 nm diameter) pleomorphic enveloped virus with a non-segmented, negative-sense, single-stranded RNA that belongs to the Pneumoviridae family and is recognised as one of the most common causes of acute respiratory tract infections in children, older people, and immunocompromised adults^14–16^. Despite the enormous burden of RSV disease, there is currently no efficacious vaccine nor antiviral drug therapy available^17^. RSV is a highly contagious pathogen and transmission is thought to be primarily by large droplets and fomites, but is yet to be fully resolved^18^. However, clinical and epidemiological studies of patients infected with RSV raised the possibility of faecal–oral transmission as described for other respiratory viruses^19–21^. Herein we used RSV as a model for potential interactions of enveloped respiratory viruses with amoebae to ascertain their possible role as an environmental reservoir and vehicle for dissemination and transmission.

Within two hours of introducing GFP-RSV to an active culture of *Willaertia magna* (co-culture) the virus was observed within trophozoites and expelled vesicles (Fig. 1a). In a separate experiment, and after 72 h post introduction, fluorescence microscopy showed expelled respirable-sized amoebal-vesicles filled with GFP-RSV (Fig. 1b). Transmission electron microscopy (TEM) revealed pleomorphic RSV particles from different cross-sections within *W. magna* phagosomes (Fig. 2a). Further to this, the presence of RSV inside purified extracellular amoebal-vesicles was confirmed by TEM (Fig. 2b). Using the ImageJ software package^22^, the virions measurements (Table 1) were consistent with previous conventional EM studies^23–25^.

**Fig. 1:**
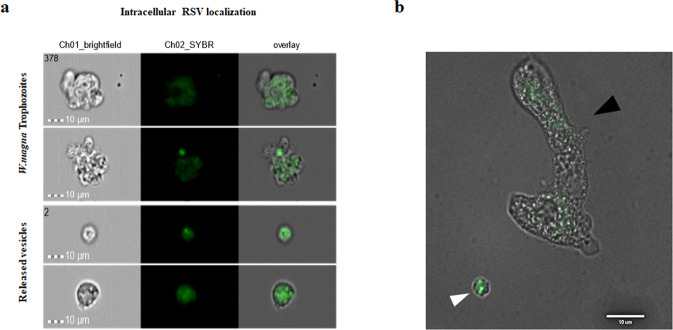
Intracellular RSV localisation. **a** Internalised GFP-RSV virus particles in live amoebae (trophozoites and released vesicles) using ImageStream^®^ flow cytometry. **b** GFP-RSV virus particles packaged in excreted vesicles by *W. magna* using fluorescent microscopy (100x), released vesicle (white arrowhead) amoeba trophozoites (black arrowhead).

**Fig. 2:**
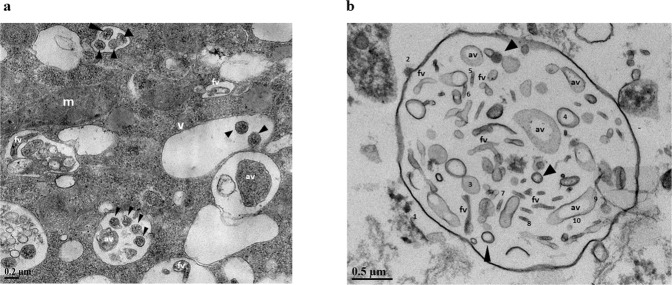
Ultrastructural visualisation of internalised RSV. **a** Transmission electron microscopy of RSV particles within *W. magna* food vacuoles after 72 h of co-culture. **b** Purified released extracellular vesicle after 24 h of co-culture containing RSV virions. Virions were randomly selected and measured using ImageJ (marked 3–10). Three morphology categories of RSV were found: spherical (black arrowheads), asymmetric (marked av), and filamentous (marked fv). mitochondria (m), vacuole (v).

**Table 1:** Extracellular Amoebal Vesicle (EAV) and internalised RSV virion measurements.

	Area	Mean	Min	Max	Angle	Length nm	Shape
EAV							
1 (width)	10344.39	172.874	24.109	240.099	0.141	2892.866	Spherical
2 (length)	10102.04	163.793	20.115	232.389	−87.973	2826.769	Spherical
RSV virions							
3	688.776	160.64	60	206.963	−128.83	187.967	Spherical
4	816.327	172.059	112	206.317	−129.226	225.905	Spherical
5	688.776	168.842	43	208.019	3.24	189.589	Spherical
6	165.816	93.058	73.75	126	−4.764	43.006	Filamentous
7	178.571	129.304	96.314	182.515	−22.62	46.429	Filamentous
8	165.816	114.662	92	150	−109.983	41.802	Filamentous
9	1785.714	185.194	125	229.638	28.775	497.096	Asymmetric
10	816.327	171.882	102.797	208.049	−61.39	223.749	Asymmetric

It is important to note, that amoebae trophozoites were visibly unaffected by the presence of internalised RSV virus.

Based on the GFP expression, it appeared that the RSV within amoebal-vesicles could still be infectious^26,27^. Therefore, it was of interest to assess the infectivity of freshly isolated RSV-EAVs (Fig. 3a). The EAVs containing RSV were collected 24 h post infection and viral titres, as measured by traditional TCID_50_ analysis, demonstrated that RSV*-*EAVs were indeed infectious with titres peaking at ~10^4^ TCID_50_ mL^−1^ (Fig. 3c), at a similar infectivity to RSV-only controls. Minor losses could be explained by the supernatant washing steps. On closer observation using phase-contrast microscopy there was also clear cytopathic effect induced by infectious RSV*-*EAVs in Hela cells, preventing the formation of the cells monolayer and affecting their appearance after 5 days of infection (Fig. 3d).

**Fig. 3:**
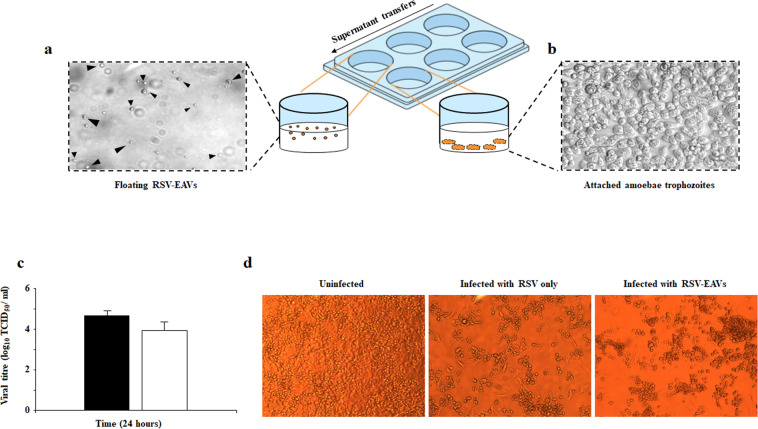
RSV-EAVs isolation and infectivity assay. Micrographs (40x) of **a** Released extracellular amoebal-vesicles containing RSV and **b** Attached amoebae trophozoites. **c** Replication kinetics of the control RSV alone (black bar) and RSV-EAVs (white bar) analysed by TCID50 on Hela cells. Data are the mean ± SEM, *n* = 3 performed in triplicate. **d** Micrographs (20x) of cytopathic effect (CPE) induced by RSV-EAVs infection in Hela cells at 5 days post-infection.

Recently, multiple independent studies have revealed that different viruses may exploit the secretory autophagy pathway to exit cells via released vesicles^28–31^. These amoebal-released packaged viruses could prolong their environmental infectivity (via fomites/aerosols/water system), as well when internalised by avoiding immune systems detection, such as evading recognition by neutralising antibodies^32^. Also, in a previous study utilising infectious Coxsackievirus B virions (i.e. a non-enveloped, enteric virus) we reported virions localised in *Vermamoeba vermiformis* trophozoites and expelled vesicles^11^. Overall, virus-laden vesicles would increase the (dose) likelihood to infect susceptible host cells^33^, as well as the virus’ infectivity, as demonstrated for enteroviruses with equivalent numbers of virions free versus within vesicles^29,34,35^. Extracellular vesicles containing enteric viruses are naturally shed in human and animal faeces (and amoebae grow in sewage/animal excreta, including bat guano)^36–38^, which could be ingested and transmit to other hosts^39^. Interestingly, as evident in Figs 1 and 2, the released amoebal-vesicles are 2–3 μm in diameter, the size range expected to penetrate to the lower respiratory tract via mouth or nose inhalation^40,41^.

Taken together these interesting observations provide evidence to suggest that amoebae may contribute to the environmental persistence and transmission of respiratory viruses associated with natural aquatic environments and engineered water systems. Notably, extracellular amoebal-vesicles could enable non-enveloped and enveloped virion dissemination and aid in the transmission of respiratory viruses. Amoeba-packaged viruses (in trophozoites, cysts and vesicles) may also protect virions from inactivation via sunlight, biocides^42^ and antiviral host factors^43,44^. Hence, we recommend further study of the persistence and transmission of respiratory viruses in faecal droplets and aerosols to assess this newly proposed risk pathway; noting that sewage droplets/aerosols were shown to be important during the first SARS epidemic^45^, and associated with toilets and COVID-19 cases in hospitals^46^. Understanding how enveloped viruses persists in our environmental systems and interact with amoebae will contribute to our understanding of the epidemiology and microbial ecology of respiratory viruses and potentially permit the development of methods to further aid in their management.

## Methods

### Strains and culture conditions

The virus used in this study was green fluorescent protein-expressing RSV (GFP-RSV) containing the viral glycoproteins (S, G and F)^47^. The RSV was propagated on 80–90% confluent HeLa cells (ATCC CCL-2) in DMEM medium containing 10% FBS, and 1% penicillin-streptomycin at 37 °C and 5% CO_2_ in vented 75 cm^2^ cell-culture flasks.

The amoebae used in this study was *Willaertia magna* (ATCC 50035), a member of the Vahlkampfiidae family that was isolated from bovine faeces. Amoebae were grown in tissue culture flasks in SCGYEM (Serum-Casein-Glucose-Yeast-Extract-Medium: ATCC medium 1021) at 25 °C in a 5% CO_2_ incubator. The trophozoites were maintained in exponential growth phase by sub-culturing every 3–4 days in fresh SCGYEM. Amoebae were harvested by tapping the flasks to dislodge surface-adhered cells and subsequent centrifugation in a 15 mL screw-cap tube (FALCON, Fischer Scientific, Edmonton, Canada 3033) at 2000 × *g* for 10 min. Cells were washed three times with sterile distilled water to remove carried-over nutrients in the supernatants.

### Imaging flow cytometry analysis

ImageStream^®^ cytometry analysis and the instrument gating strategy for amoebae was performed as previously described^37^. Briefly, *W. magna* trophozoites were infected for 2 h with GFP-RSV at MOI of 100, washed and re-suspended in PBS prior to processing through the ImageStream^®^X Mark II (Millipore Sigma). Cells were examined at 60× magnification. Analysis was performed using the IDEAS software (Amnis, Seattle) and cells (fluorescent viruses and amoebae) were identified on the basis of bright field morphology, size and GFP signal.

### Isolation of extracellular amoebal-vesicles (EAVs) containing RSV

*W. magna* and RSV were co-cultured at a ratio of 1:100 in conical Falcon tubes containing 3 mL of SCGYEM medium, vortexed to favour virus interaction with amoebae and then transferred to 6-well culture plates (Fisher Scientific 130185). After overnight incubation at 30 °C, samples were analysed using a phase-contrast microscope (Leica CTR 4000) to detect the presence of EAVs in the supernatant while amoebal trophozoites remain attached to the surface of the well plates. To isolate and separate the EAVs containing RSV from the attached trophozoites, supernatants were removed and transferred into new well plates several times. In brief, supernatants were gently removed with care taken not to disturb the attached amoebae on well plate surfaces, and transferred to new well plates for 10–20 min to allow any amoebal trophozoites to attach to surfaces (Fig. 3b). The isolated EAVs containing RSV were collected and washed twice with PBS by centrifugation at 4000 × *g* for 5 min to remove uninternalized viruses. The purified EAVs were then used for infectivity assays and microscopy.

### RSV infectivity assays

RSV was released from amoebal vesicles by three consecutive freeze−thaw cycles. RSV infectivity (EAVs containing RSV and RSV-only control) was measured by infecting confluent HeLa cells in quadruplicate using 48-well plates and serial dilution of the virus in HeLa cells maintenance medium. Cells were observed daily for cytopathic effects for seven days and CPE was measured by the tissue culture infectious dose 50% (TCID_50_) using the Reed–Muench formula^48^.

### Transmission electron microscopy

Axenic cultures of *W. magna* were co-cultured with RSV at a MOI of 100 on Thermonax^®^ cover slips (Thermo Fisher 174985). After decanting the medium, amoebae were fixed at room temperature with 2.5% glutaraldehyde and 0.1 M sodium cacodylate buffer (Electron Microscopy Sciences 15960). The samples were submitted for processing at the imaging core at University of Alberta, faculty of biological sciences. Sectioned and carbon-coated samples were observed with a Hitachi H-7650 transmission electron microscope.

### Fluorescence microscopy

Co-cultures of *W. magna*-*GFP-RSV* were carried in 12-well tissue culture plates overlaid with microscopy cover slips (Fisher Scientific 12-5461) and incubated at 25 °C with 5% CO_2_. After 72 h of infection, the medium was removed and cells fixed with 4% paraformaldehyde for 5 min at room temperature and then washed with phosphate-buffered saline three times. Images were taken with an EVOS FL fluorescent cell imaging system (ThermoFisher Scientific).

### Reporting summary

Further information on research design is available in the Nature Research Reporting Summary linked to this article.

## Supplementary information

Reporting Summary

## Data Availability

The data sets generated during and/or analysed during the current study are either shown in the manuscript or available from the corresponding author on reasonable request.
